# Hypothalamus-Related Resting Brain Network Underlying Short-Term Acupuncture Treatment in Primary Hypertension

**DOI:** 10.1155/2013/808971

**Published:** 2013-05-27

**Authors:** Hongyan Chen, Jianping Dai, Xiaozhe Zhang, Kai Wang, Shuhua Huang, Qingtian Cao, Hong Wang, Yuhong Liang, Chuanying Shi, Mengyuan Li, Tingting Ha, Lin Ai, Shaowu Li, Jun Ma, Wenjuan Wei, Youbo You, Zhenyu Liu, Jie Tian, Lijun Bai

**Affiliations:** ^1^Department of Radiology, Beijing Tiantan Hospital Affiliated to Capital Medical University, Tiantan Xili No. 6, Beijing 100050, China; ^2^Beijing Neurosurgery Institute, Tiantan Xili No. 6, Beijing 100050, China; ^3^Department of Pain, Beijing Tiantan Hospital Affiliated to Capital Medical University, Tiantan Xili No. 6, Beijing 100050, China; ^4^Department of Medicine, China North Vehicle Research Institute Worker's Hospital, Huaishuling No. 4, Fengtai District, Beijing 100072, China; ^5^Tiantan Community Health Service Center, Fenchang Hutong No. 57, Dongcheng District, Beijing 100061, China; ^6^Ultrasonic Center, Beijing Tiantan Hospital Affiliated to Capital Medical University, Tiantan Xili No. 6, Beijing 100050, China; ^7^Institute of Automation, Chinese Academy of Sciences, Beijing 100190, China; ^8^The Key Laboratory of Biomedical Information Engineering of Ministry of Education, Department of Biomedical Engineering, School of Life Science and Technology, Xi'an Jiaotong University, Xi'an 710049, China

## Abstract

The present study attempted to explore modulated hypothalamus-seeded resting brain network underlying the cardiovascular system in primary hypertensive patients after short-term acupuncture treatment. Thirty right-handed patients (14 male) were divided randomly into acupuncture and control groups. The acupuncture group received a continuous five-day acupuncture treatment and undertook three resting-state fMRI scans and 24-hour ambulatory blood pressure monitoring (ABPM) as well as SF-36 questionnaires before, after, and one month after acupuncture treatment. The control group undertook fMRI scans and 24-hour ABPM. For verum acupuncture, average blood pressure (BP) and heart rate (HR) decreased after treatment but showed no statistical differences. There were no significant differences in BP and HR between the acupuncture and control groups. Notably, SF-36 indicated that bodily pain (*P* = 0.005) decreased and vitality (*P* = 0.036) increased after acupuncture compared to the baseline. The hypothalamus-related brain network showed increased functional connectivity with the medulla, brainstem, cerebellum, limbic system, thalamus, and frontal lobes. In conclusion, short-term acupuncture did not decrease BP significantly but appeared to improve body pain and vitality. Acupuncture may regulate the cardiovascular system through a complicated brain network from the cortical level, the hypothalamus, and the brainstem.

## 1. Introduction

Hypertension is a common chronic disease affecting one-third of all adults worldwide and causing 51% of stroke deaths and 45% of coronary heart disease deaths in 2012 [1]. Investigating hypertension relief has therefore gained increasing attention and interest.

Acupuncture has emerged as a common alternative or complementary therapeutic intervention in western medicine. Reports have stated that acupuncture has certain curative effects for high BP and cardiac pain, with few side effects observed [2]. Despite its public acceptance, unequivocal scientific explanations regarding the mechanism underlying acupuncture in high BP treatment has not been attained and awaits further investigation.

In the recent years, the therapeutic effect of acupuncture in lowering BP has been investigated and discussed in many case reports and small-scale clinical trials [3–7], as well as several large-scale clinical trials on hypertension with acupuncture treatments [8–10]. However, these studies have only focused on the efficacy of acupuncture, and the mechanism of acupuncture in hypertension relief remains unknown.

In the past two decades, converging evidence from fMRI studies has demonstrated that acupuncture stimulation can modulate neural activities in a wide cortical-subcortical network, particularly the limbic system [11–19]. In practice, the well-identified physical effects of acupuncture and its purported clinical efficacy also suggest that it helps maintain a homeostatic balance of the internal state within and across multiple brain systems. Evidence from animal studies has demonstrated that acupuncture stimulation can facilitate the release of certain neuropeptides in the central nervous system (CNS), eliciting profound physiological effects and even activating self-healing mechanisms [20, 21]. Electroacupuncture studies in rats revealed that both low-frequency and high-frequency stimulation induced analgesia, but differential effects existed in low- and high-frequency acupuncture on the types of endorphins released [20]. Peripheral acupuncture stimulation in deeper areas also activated various brain structures, such as the limbic, hypothalamic, and brainstem neural nuclei [21].

The present study attempted to investigate short-term acupuncture treatment in hypertension relief using resting state fMRI combined with a 24-hour ABPM. The aim was to (i) observe if short-term acupuncture decreased BP of hypertensive patients and improved quality of life and (ii) determine the possible mechanism of short-term acupuncture for treating hypertension in the hypothalamus-related brain network.

## 2. Materials and Methods

### 2.1. Participants

We recruited hypertensive participants from the Beijing Dongcheng District Tiantan community health service center and Fengtai District 201 community hospital. The eligibility criteria included (1) clinical diagnosis in line with WHO diagnostic criteria of essential hypertension: systolic blood pressure (SBP) ≥140 mmHg and diastolic blood pressure (DBP) ≥90 mmHg without antihypertensive drugs, two or more repeated measurements, normal BP now with the use of antihypertensive drugs but with a clear history of hypertension, and species and dose of antihypertensive medicine not changed or suspended or not taking antihypertensive drugs during the study period [8, 9]; (2) 30–75 years old; (3) without cerebral infarction history and without cerebral infarction by MRI preliminary screening; (4) without nervous system disease, mental disease, diabetes, or other serious illness; (5) no drug addiction history; (6) no MRI contraindication and acupuncture contraindication; (7) no acupuncture experience over the past year; (8) right-handed by Edinburgh handedness questionnaire [19]; (9) provision of informed consent. The exclusion criteria included (1) could not receive acupuncture; (2) any unsuitable situation in the study. The drop-off criteria are as follows: (1) hypertensive crisis or other emergency; (2) could not adhere to acupuncture treatment. The present study was according to the principles of the Declaration of Helsinki (Version Edinburgh 2000).

**Figure 1:**
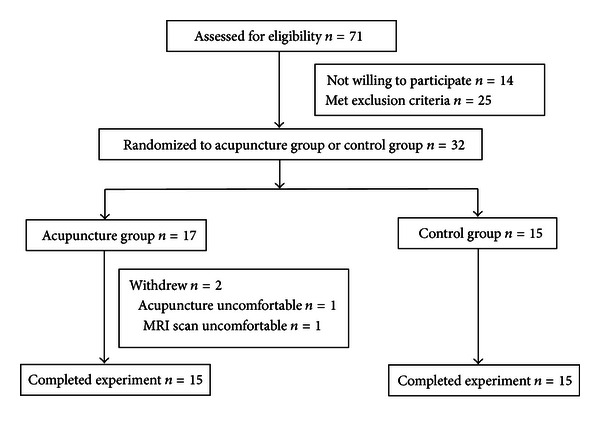
Participants flowchart diagram.

Of the 71 participants recruited, only 30 (14 male, 35–74 years, mean age of 56.73 ± 9.29 years) met the eligibility criteria and finished the experiment (Figure 1). All subjects were randomly divided into the acupuncture group and the control group, with 15 participants in each.

### 2.2. Acupuncture Interventions

**Figure 2:**
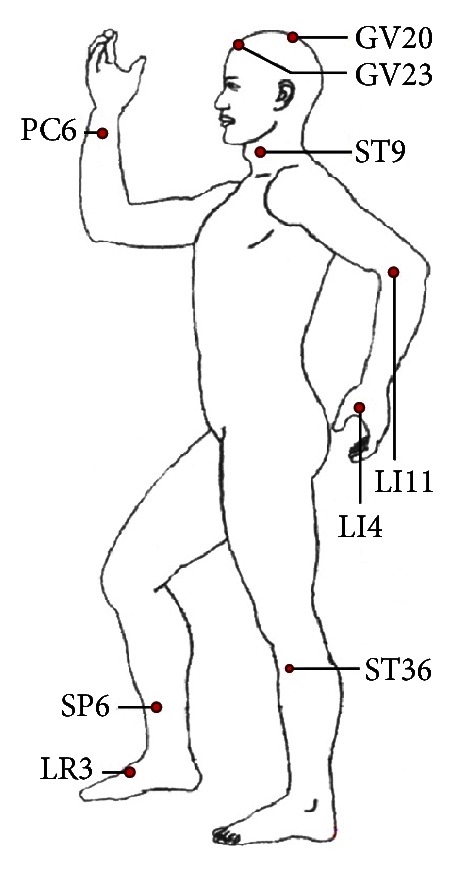


**Figure 3:**
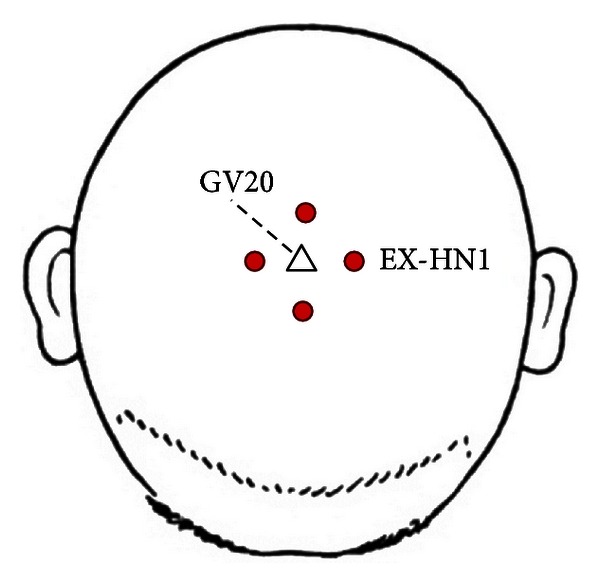


Participants underwent five acupuncture treatments over five consecutive days, with each session lasting about 30 minutes. During each treatment, seven acupoints used to treat hypertension in clinical settings were selected and needled bilaterally (except single points like GV20 (baihui), GV23 (shangxing), and four points EX-HN1 (sishencong)), including ST9 (renying), LI11 (quchi), PC6 (neiguan), LI4 (hegu), ST36 (zusanli), SP6 (sanyinjiao), and LR3 (taichong) [10, 22] (Figures 2 and 3). After local skin disinfection, sterile acupuncture needles (0.2 mm in diameter, 40 mm long, Hua tuo acupuncture needles, Suzhou, China) were inserted into the skin to a depth of 15–50 mm according to different acupoints and were gently twisted in a mild reinforcing-reducing method 4–6 times till a *deqi* response was obtained. Control group participants did not receive acupuncture treatment and took medicine according to their original treatment programs. The acupuncture procedure was performed by the same experienced and licensed acupuncturist (15 years of experience) on all subjects.

Figures 2 and 3 show needling acupoints for hypertension treatment. GV20 (baihui) is located in the center of the head; EX-HN1 (sishencong) includes four acupoints located around GV20, each about 3 cm to GV20; ST9 (renying) is located in the neck, beside the Adam's apple, in the midpoint of the leading edge of the sternocleidomastoid; LI11 (quchi) is located in the outer end of the elbow stripes; LI4 (hegu) is located in the back of the hand, between the first and second metacarpal, radial side of the midpoint of the second metacarpal; PC6 (neiguan) is located in the volar forearm, about 6 cm above the wrist stripes between tendon and radial measured wrist flexor tendon; ST36 (zusanli) is located in the anterolateral leg, between the tibialis anterior muscle and extensor digitorum longus, about 10 cm below the knee, about 1.5 cm from the tibia leading edge; SP6 (sanyinjiao) is located in the medial leg, about 10 cm above the medial condyle tip; LR3 (taichong) is located in the dorsal foot and the first metatarsal gap rear depression.

### 2.3. Data Acquisition

The participants were scanned in a 3.0 Tesla Siemens Trio MR whole body scanner. A foam pillow and a band across the forehead were used to fix the head. Resting state functional images were acquired with a single-shot gradient recalled echo planar imaging sequence. The sequence covered the whole brain, axial view, parallel to the AC-PC line, TR = 2000 ms, TE = 30 ms, measurement = 240, resolution = 64 × 64, field of view (FOV) = 240 mm × 240 mm, flip angle = 90°, slice thickness = 5 mm without gap, 32 slices, and scan time = 8,06 minutes. A set of T1-weighted high-resolution structural images were collected using a 3D MPRAGE sequence for anatomical localization. TR = 1900 ms, TE = 2.39 ms, field of view (FOV) = 256 mm × 256 mm, flip angle = 7°, in-plane resolution = 1 mm × 1 mm, slice thickness: 1 mm without gap, 32 slices, and scan time = 8,26 minutes.

### 2.4. Experiment Workflow

The experiment workflow is shown in Figure 3. The first MRI scan adopted the T2WI sequence to exclude participants with cerebral infarction. Participants who met the inclusion criteria underwent resting state fMRI and 3D T1WI structure sequences. The second and third MRI scans were only performed in the resting state fMRI sequence. The BP and heart rate (HR) of the participants were measured three times before and after every MRI scan, with the average taken for the three measurements. Participants sat quietly for 5 minutes before each measurement. We used a MOBIL GRAPH sphygmomanometer (Germany) to monitor 24-hour ambulatory blood pressure (ABMP). The BP and HR of the participants were also tested three times before and after every acupuncture treatment, with the average taken for the three recordings.

The acupuncture group received 24-hour ABMP and an MRI scan at multiple time points (before acupuncture, after acupuncture, and one month later). The control group only received 24-hour ABMP and one MRI scan. During the study period, participants maintained their original treatments, and drug type and dosage were not modified except for emergency situations (SBP ≥ 180 mmHg, DBP ≥ 110 mmHg, or other emergency situations). In case of emergency, the participants were moved into the drop-off group.

At the end of the first acupuncture treatment, the participants completed a questionnaire using a 10-point visual analog scale (VAS) to rate the experience of *deqi* during intervention. The questionnaire included aching, pressure, heaviness, coolness, soreness, fullness, numbness, warmth, tingling, dull pain, and sharp pain. The VAS was defined as 0 = no sensation, 1–3 = mild, 4–6 = moderate, 7-8 = strong, 9 = severe, and 10 = unbeatable sensation.

**Figure 4:**
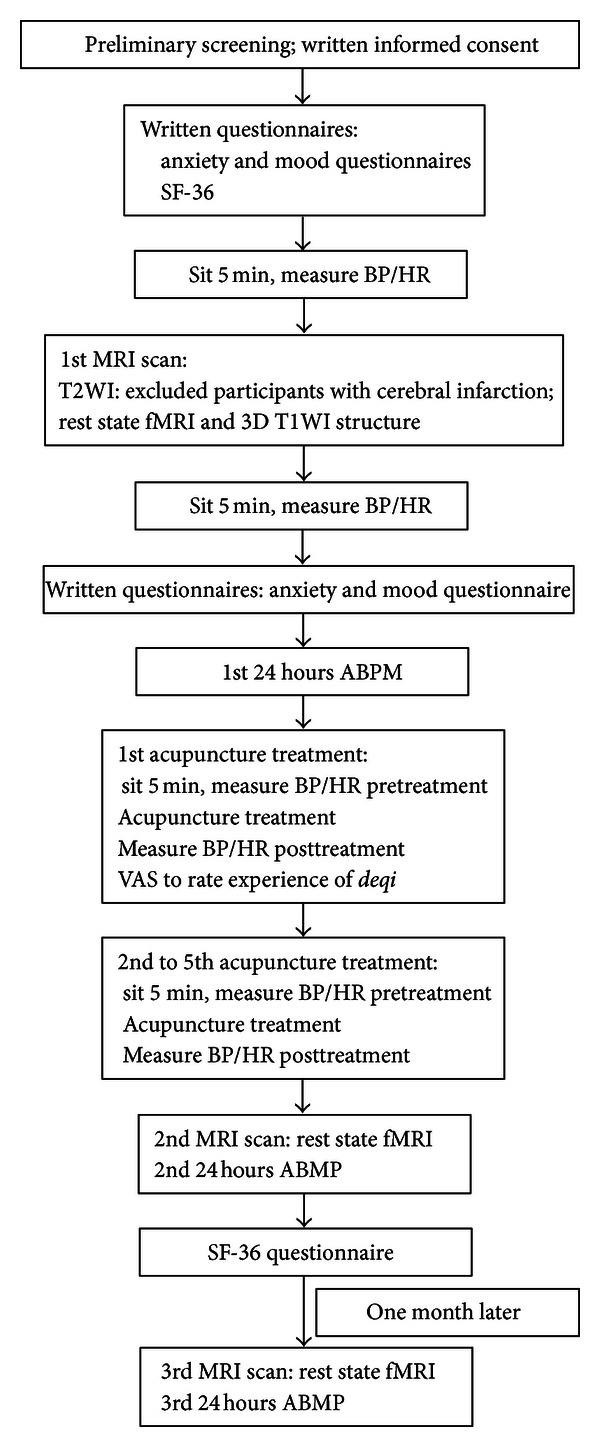
Experimental workflow.

Considering that BP and HR can be easily affected by psychological factors, such as affective states and anxiety, participants filled out questionnaires for assessments of anxiety (State Trait Anxiety Inventory (STAI)) [23] and affective state (BFS mood survey) [24]. To evaluate quality of life (QoL), subjects also completed questionnaires on MOS item short from health survey (SF-36) [25] before and after acupuncture treatment in the acupuncture group. The control group participants also completed the SF-36 (see Figure 4).

### 2.5. Statistical Analysis

#### 2.5.1. Physiological Data

Since 24-hour ABMP is affected by movement and other factors, the data collected from each participate was not exactly the same. Therefore, we selected independent sample *t*-tests to compare the physiological data and paired *t*-tests to analyze the SF-36 survey. We used SPSS 13.0 for statistical analyses, with *P* < 0.05 indicating statistical difference and *P* < 0.01 indicating significant statistical difference.

#### 2.5.2. Image Preprocessing

All preprocessing steps were carried out using statistical parametric mapping (SPM5, http://www.fil.ion.ucl.ac.uk/spm/). Functional images were preprocessed using sinc interpolation for slice scan time correction, trilinear sinc interpolation for alignment (motion correction) of functional volumes, and high-pass temporal filtering to 1 Hz to remove slow drifts in the data. The image data were further processed with spatial normalization based on the MNI space and resampled at 2 mm × 2 mm × 2 mm. Poststimuli resting data were also filtered using a band pass filter (0.01~0.08 Hz) to reduce low-frequency drift and high-frequency noise. Finally, the functional images were spatially smoothed with a 6 mm full width at half maximum (FWHM) Gaussian kernel. All resting state functional images were preprocessed using Statistical Parametric Mapping 5 (SPM5) and included motion correction, normalization, and smoothing.

#### 2.5.3. Functional Connectivity Analysis

For each subject, the “seeding” time courses of the hypothalamus were, respectively, cross-correlated with all low-pass filtered voxels to generate functional connectivity maps within each of the three conditions. This approach was termed within-condition interregional covariance analysis (WICA). The resulting correlation coefficient *r*-maps were normalized and corrected to roughly standard normal distributions using methods previously described. The normality of the distribution was then tested using Kurtosis tests (*P* < 0.001). The three *z*-maps of each individual were entered into one-sample *t*-tests, respectively, to determine whether group data was significantly different from zero. For visualization, all connectivity results were transformed into the Talairach stereotactic space and overlaid on MRIcro (http://www.mccauslandcenter.sc.edu/CRNL/) for presentation purposes. All resulting *t*-maps were then cluster-filtered to remove correlations involving less than three contiguous voxels and then superimposed on high-resolution anatomical images using a *P* < 0.001 cutoff threshold (uncorrected). The above image processing programs were coded in MATLAB7 (MathWorks, Inc.).

## 3. Results

### 3.1. Physiological Data

Average BP at different time points for the acupuncture and control groups are shown in Table 1. Two independent sample *t*-tests were used to compare the BP and HR between the two groups, *t* = −1.529, *P* = 0.127, with no statistical differences observed in physiological data, indicating comparable balance in the two groups.

Comparison between acupuncture treatment before and after showed that the average SBP, DBP, and HR tended to decrease after acupuncture, although no statistical differences were observed (Table 2). Comparison between the acupuncture and control group showed that average SBP, DBP, and HR demonstrated no statistical differences between the two groups (Table 3).

In the SF-36 survey for QoL, participants in the acupuncture group reported increased scores of bodily pain (baseline: mean ± SD, 79.4 ± 12.3 versus after acupuncture: mean ± SD, 87.0 ± 10.6, *P* = 0.005) and vitality (80.0 ± 15.9 versus 83.2 ± 11.9, *P* = 0.036). Changes in physical functioning (92.7 ± 11.9 versus 92.7 ± 11.9), role physical (76.7 ± 38.3 versus 78.3 ± 35.2, *P* = 0.582), general health (67.0 ± 15.2 versus 68.7 ± 13.9, *P* = 0.334), social functioning (96.7 ±  23.36 versus 98.3 ± 19.4, *P* = 0.334), role emotional (91.1 ± 26.6 versus 93.3 ± 25.8, *P* = 0.334), and mental health (70.4 ± 12.9 versus 71.2 ± 12.5, *P* = 0.082) did not differ among participants.

### 3.2. Connectivity Mapping

At the baseline, the hypothalamus showed prominently spontaneous activations associated with limbic, cortical, and subcortical regions (*P* < 0.001), including the bilateral cerebellum, middle brain, bilateral insula, thalamus, and most of the frontal lobes. These results demonstrated a hypothalamus-anchored resting brain network under baseline conditions. Conversely, spontaneous deactivation was mainly located in the left cerebella vermis, as well as the left superior and right inferior frontal gyrus.

**Figure 5:**
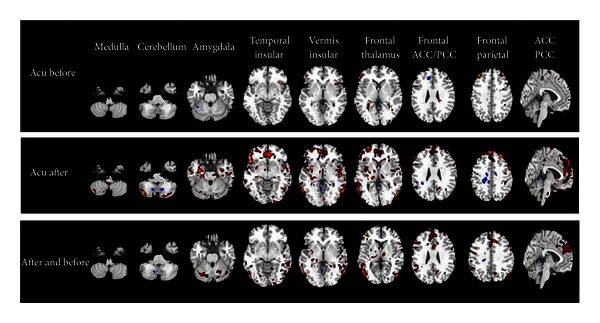
Functional connectivity anchored with the hypothalamus after and before acupuncture treatment.

After acupuncture treatment, these spontaneous activation and deactivation networks anchored by the hypothalamus remained relatively stable (Figure 5, *P* < 0.001). In comparison with baseline conditions, however, we also identified significant changes (both in spatial distributions and response magnitudes) after acupuncture treatment. There were prominently increased spontaneous activations in the bilateral cerebellum, brainstem, limbic system (bilateral insula, hippocampus, amygdala, and cingulate cortex), bilateral thalamus, and bilateral frontal lobes. Enhanced deactivations were located in the bilateral cerebellum, bilateral frontal lobes, and right parietal gyrus. 

**Figure 6:**
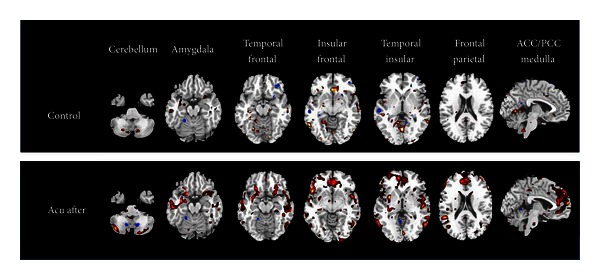
Functional connectivity anchored with the hypothalamus resulting from acupuncture control.

Results from after and before acupuncture showed a wide range activation of brain regions (Figure 5). Notably, the most modulated changes were exhibited in the cerebellum, brainstem, insula, and frontal lobe. Increased positive correlations were primarily located in the cerebellum, limbic system (insula, parahippocampal gyrus, and cingulate cortex), bilateral thalamus, and frontal lobes. It is also worth noting that enhanced deactivation was only identified in the temporal lobe, left posterior cingulum, and right parietal lobe. Results from the acupuncture and control groups also showed significant differences in the cerebellum, amygdala, brain stem, and insula, and frontal lobes (see Figure 6).

## 4. Discussion

The present short-term acupuncture intervention study used common acupoints to clinically treat hypertension once a day over five consecutive days. After acupuncture, no statistical differences were found in SBP, DBP, and HR between the before acupuncture and control groups. However, average SBP, DBP, and HR did decrease after acupuncture treatment. The SF-36 survey from the acupuncture group showed statistical differences in body pain and vitality after acupuncture compared to the baseline. Our results did not indicate any differential benefit of short-term acupuncture for controlling BP and HR, but it may improve quality of life for patients in relation to body pain and vitality. This is the first study to investigate the hypothalamus-anchored brain network involving acupuncture treatments for hypertension.

Several large scale hypertension studies have investigated and discussed the therapeutic effects of acupuncture treatment. Flachskampf et al. [8] randomized 160 hypertensive participants in a single-blind 6-week trial using active or sham acupuncture and found significant (*P* < 0.001) differences in posttreatment BP between the two groups. Yin et al. [9] recruited 41 hypertensive or prehypertensive patients and designed a randomized, double-blind, placebo-controlled trial. All subjects were randomly assigned into real or sham acupuncture groups, and after 8 weeks of intervention, the mean BP of the acupunctured group was significantly decreased (*P* < 0.01). The Stop Hypertension with Acupuncture Research Program (SHARP) [10] showed there were no significant differences in BP decrease in the individualized traditional Chinese acupuncture group (IND), standardized acupuncture group (STD), and invasive sham acupuncture group (CNTL). Despite there being no statistically significant differences, mean decreases in BP after 10 weeks of treatment were observed between active (IND and STD) and sham acupuncture in comparison to the baseline in the SHARP study. The trials of Flachskampf et al. [8] and Yin et al. [9] showed a decrease in BP after acupuncture, but SHARP [10] only showed a downtrend without statistical differences. In previous studies, we found that the Flachskampf's and Yin's studies were long term with intensive frequency of acupuncture treatment, while the SHARP study was long term with infrequent acupuncture treatment. Acupuncture in our study was short term and intensive, which resulted in no significant differences between acupuncture and control group. Considering that acupuncture has sustained and cumulative effects, short term use may lead to inconsistent results. We therefore speculated that the efficacy of frequent, long-term acupuncture would be better than long interval use or short-term treatment in hypertensive patients.

The cardiovascular center is mainly located in the medulla oblongata and hypothalamus, and these areas are not in the default brain network [26]. Many electrophysiological studies [27–31] have shown that the hypothalamus may be a key hub in the CNS in relation to cardiovascular regulation. Thus, we selected the hypothalamus as the seed point region to explore modulated brain network changes underlying acupuncture hypertension treatment. Our results suggested that increased functional connectivity existed between the hypothalamus and other regions after acupuncture. The functional connection with the hypothalamus was enhanced in some areas, mainly in the bilateral frontal lobe (vmPFC, dlPFC), limbic system (bilateral insula, amygdala, hippocampus, anterior cingulate cortex, and posterior cingulate cortex), bilateral cerebellum, and medulla oblongata. The central regulatory region of the cardiovascular system is very wide from brainstem to cortex, and includes the medulla oblongata, pons (locus coeruleus), midbrain (periaqueductal gray, substantia nigra), and limbic system (hypothalamus, the amygdaloid, subfornical organ, and insula), which together make up a complete network [27]. The medulla oblongata is the basic center, which includes the rostral ventrolateral medulla (vasoconstriction), ventrolateral part of the medulla oblongata (vasodilation), ambiguous nucleus and dorsal nucleus of the vagus nerve (restrains heart function), and tractus solitarius medulla oblongata (afferent nerve terminal) [28]. Past research has indicated that the medulla oblongata regulates the cardiovascular system through neurotransmitters such as glutamate, P substance and *γ*-aminobutyric acid [29–31].

Previous studies on electrophysiology have shown that the periaqueductal gray (PAG), hypothalamus, and other parts of the limbic system were important parts of the cardiovascular center [32–40] through different neurotransmitters, adjusting the sympathovagal balance to regulate the cardiovascular system. Many projection fibers are found between the ventrolateral medulla, PAG, and hypothalamus arcuate nucleus for their closely interconnected, and adjusted the cardiovascular system in common [32–34]. The limbic system also plays an important part in BP regulation in the CNS. Some parts are involved in step-up regulation, such as the paraventricular nucleus, lateral hypothalamus/perifornical area, dorsomedial nucleus, ventromedial nucleus, posterior hypothalamic nucleus, lateral septum ventrolateral part/habenular body, subfornical organ, and the central amygdala and insula, while the anteroventral third ventricle area (AV3V area) and the arcuate nucleus participate in decompression regulator, with interactions occurring between these regions [28, 29, 35–40]. Although the role of the cerebellum in cardiovascular regulation is unclear, animal experiments suggest that the neuronal excitability of the fastigial nucleus has the effect of decreasing BP [41–43]. Electrical stimulation of the fastigial nucleus can increase arterial BP and HR, which relieve neuron injury through chronic cerebral ischemia, and was helpful for damaged arterial baroreflex function recovery [41–43]. Normal adults can also present transient changes in physiological indicators after fastigial nucleus electrical stimulation, which suggests a decrease in peripheral vascular flow and an increase in peripheral resistance and a slight increase in heart rate [44]. In addition, the vermiform process of the cerebellum may be important in cerebellum cycle control [45]. Idiaquez et al. [46] reported on vermis and right cerebellar hemisphere resection from cerebellar hemorrhage, where the patient appeared transient orthostatic hypotensive, suggesting that the cerebellum was involved in cardiovascular regulation. We found a negative correlation to the hypothalamus in the fastigial nucleus, which may play a part in the therapeutic action of acupuncture.

Beissner et al. [47] combined cardiac-gated brainstem-sensitive fMRI to investigate the mechanism of acupuncture. They found that, from the cortical level, the fMRI group showed significant increases activation in dorsolateral prefrontal gyrus (dlPFC), anterior mid cingulated cortex (ACC), insula, and frontal lobe cortex. Deactivations were found in the ventromedial prefrontal and orbitofrontal cortices (vmPFC/pgACC). They supposed that these areas were involved in acupuncture-induced heart rate changes. Further, as these areas are typical cortical and brainstem centers of pain and autonomic processing, they hypothesized that acupuncture may be a low-intensity deep pain stimulus that can activate autonomic concomitants and exert nonanalgesic effects with therapeutic potential. Our results also indicated a positive correlation with the hypothalamus in vmPFC, ACC, insula, and dlPFC, and these areas may participate in cardiovascular regulation in acupuncture treatment of hypertension and relieve patient pain.

The present study was limited by the small sample size. During our research, patients were found to have different reactions to acupuncture, with some being sensitive to acupuncture and some not. A larger sample size would be necessary to draw more definitive conclusions. Secondly, clinical experience and comparison with large trials indicated that long-term, frequent acupuncture may have good efficacy. The present study indicated that short-term acupuncture did not improve the BP of primary hypertensive patients. Finally, nonbrainstem sensitive fMRI sequences were used here. Because the main cardiovascular center is located in the brainstem, a more brainstem sensitive sequence is needed to investigate the mechanism of acupuncture for treating hypertension.

## 5. Conclusions

According to our results, short-term acupuncture did not decrease BP significantly but may have improved body pain and vitality. Acupuncture may also regulate the cardiovascular system through a complicated brain network from the cortical level, the hypothalamus, and the brainstem. However, the mechanism remains unclear and needs further investigation.

## Figures and Tables

**Table 1:** Physiological data of acupuncture and control groups.

	SBP (mmHg)	SD	DBP (mmHg)	SD	HR (bpm)	SD
ACU						
B	127.13	5.52	81.71	8.40	71.50	8.67
A	126.74	7.93	80.86	13.36	69.83	9.53
L	123.22	9.47	81.11	16.23	69.98	9.60
CON	133.40	18.58	80.93	11.49	72.71	6.43

ACU: acupuncture group; CON: control group; B: before acupuncture; A: after acupuncture; L: one month later.

**Table 2:** Physiological data comparison of acupuncture group (independent *t*-test).

	SBP				DBP				HR			
	*t* value	*P* value	95% CI		*t* value	*P* value	95% CI		*t* value	*P* value	95% CI	
B-A	0.453	0.658	−5.090	3.327	1.194	0.254	−2.967	0.855	1.647	0.124	−0.493	3.657
B-L	1.278	0.242	−3.321	11.138	0.008	0.994	−5.871	5.910	1.021	0.341	−1.996	5.029
A-L	0.935	0.361	0.609	6.992	−0.802	0.449	−16.831	8.307	−0.119	0.908	−3.227	2.917

B: before acupuncture; A: after acupuncture; L: one month later.

**Table 3:** Physiological data comparisons between acupuncture and control group (independent *t*-test).

	SBP				DBP				HR			
	*t* value	*P* value	95% CI		*t* value	*P* value	95% CI		*t* value	*P* value	95% CI	
B-C	−1.900	0.076	−19.976	1.118	0.207	0.838	−7.031	8.602	−0.074	0.941	−6.144	5.715
A-C	−1.646	0.117	−19.451	2.360	0.453	0.655	−6.521	10.205	−0.656	0.517	−7.422	3.830
L-C	−1.774	0.091	−26.264	2.126	0.030	0.976	−12.157	12.511	−0.802	0.432	−9.837	4.376

B: before acupuncture; A: after acupuncture; L: one month later; C: control group.
